# Long-term ion release, fluoride recharge, pH modulation, and mechanical aging of an experimental ACP-based composite compared with contemporary bioactive restorative materials

**DOI:** 10.1016/j.jobcr.2026.101431

**Published:** 2026-03-12

**Authors:** Anshuman Shetty, Mohammed Abdul Saleem, Ameer Akhil Ahmed Shaik, Mohammed Mustafa, Rahul Tiwari, Tanisha Shetty

**Affiliations:** a Department of Conservative Dentistry and Endodontics, NITTE (Deemed to be University) AB Shetty Memorial Institute of Dental Sciences Deralakatte, Mangaluru, Karnataka, 575018, India; b Department of Oral and Maxillofacial Surgery, Naseem Jeddah Medical Center, Al Oudabaa, Aziziyah, Jeddah, 23342, Saudi Arabia; c Narayana Dental College & Hospital, Nellore, Andhra Pradesh, 524003, India; d Department of Conservative Dental Sciences, College of Dentistry, Prince Sattam Bin Abdulaziz University, Al-Kharj, 11942, Saudi Arabia; e Department of Dental Research Cell, Dr. D. Y. Patil Dental College and Hospital, Dr. D. Y. Patil Vidyapeeth (Deemed to be University). Pimpri, Pune, 411018, India

**Keywords:** Dental composites, Bioactivity, Fluoride recharge, Calcium phosphate nanoparticles, Mechanical properties

## Abstract

**Aim:**

This study evaluated the 12-month ion release, fluoride recharge capacity, pH modulation, mechanical aging, and structural reliability of an experimental amorphous calcium phosphate (ACP)-based composite compared to contemporary bioactive restorative materials.

**Methods:**

An experimental ACP composite was compared with Activa™ BioACTIVE-Restorative, Cention N, and Surefil one ™. Calcium (Ca^2+^), phosphate (PO_4_^3−^), and fluoride (F^−^) release, along with pH changes, were assessed over 365 days in artificial saliva. Fluoride recharge was performed at six months using a single application of 5000 ppm sodium fluoride gel, followed by cumulative fluoride re-release measurement for 30 days. The flexural strength and modulus were evaluated at baseline, 6 months, and 12 months. Statistical analysis was conducted using mixed-model ANOVA with post-hoc tests, and flexural strength reliability was assessed using Weibull analysis (α = 0.05).

**Results:**

All materials exhibited an initial burst of ion release, followed by a progressive decline (p = 0.001). The ACP composite demonstrated the highest early calcium and phosphate release but negligible fluoride release and significant reductions in mechanical properties and reliability after aging. Cention N showed sustained multi-ion release, the greatest alkalizing capacity, and stable mechanical performance. Surefil one™ achieved the highest fluoride release and recharge capacity while maintaining a near-neutral pH and superior mechanical reliability. The Activa™ BioACTIVE-Restorative exhibited moderate ion release and intermediate mechanical performance.

**Conclusions:**

Bioactive restorative materials exhibit distinct material-dependent behaviors. Alkasite-based systems showed balanced ion release and mechanical stability, fluoride-focused systems demonstrated superior recharge and reliability, and the ACP composite provided primarily short-term calcium–phosphate release with limited long-term durability.

## 1 Introduction

The longevity of direct posterior restorations continues to be influenced predominantly by secondary caries and material degradation, which remain the most common causes of restoration failure in clinical practice.^1^ Conventional resin composites, although mechanically robust, lack the ability to modulate the oral environment or provide sustained therapeutic ion release. This limitation has driven the evolution of bioactive restorative materials that aim not only to restore tooth structure, but also to contribute to preventive functions by releasing calcium, phosphate, and fluoride ions, neutralizing acidic challenges, and supporting remineralization.^2^^,^^3^

In recent years, the concept of bioactivity in resin-based materials has increasingly focused on the incorporation of ion-releasing fillers at the micro- and nano-scales. Calcium phosphate nanoparticles have attracted considerable attention because of their high surface area, enhanced solubility, and ability to release biologically relevant ions under cariogenic conditions. Nanoparticle-based systems are expected to provide more controlled and sustained ion release than conventional glass fillers, potentially improving the long-term remineralization performance.^4^

Amorphous calcium phosphate (ACP) is particularly promising owing to its thermodynamic instability and rapid dissolution in acidic environments, which results in the release of calcium and phosphate ions that can promote apatite formation. Previous studies have demonstrated that ACP-containing composites and sealants can provide significant initial ion release and remineralization potential. However, these materials often exhibit a pronounced burst release effect followed by rapid depletion, raising concerns regarding their long-term bioactivity and mechanical durability.^5^^,^^6^

Commercially available bioactive systems such as Activa™ BioACTIVE-Restorative, Cention N, and Surefil one™ represent different contemporary approaches to combining bioactivity with clinical practicality.^7^, ^8^, ^9^ Activa™ BioACTIVE-Restorative incorporates a resin-modified glass-ionomer, resin hybrid matrix designed to release calcium, phosphate, and fluoride.^7^ Cention N is an “alkasite” restorative that contains alkaline fillers capable of modulating pH and delivering sustained fluoride release.^9^ Surefil one™ merges resin-composite technology with glass-ionomer chemistry, allowing dual acid–base and polymerization reactions intended to enhance fluoride release and reduce technique sensitivity.^8^ Despite the increasing availability of such materials, the term “bioactive” encompasses systems with widely different ion-release mechanisms, filler chemistries, and clinical behaviors. As a result, direct comparisons among different classes of bioactive materials remain limited, and the long-term clinical relevance of their ion release profiles is still debated.

Several studies have indicated that many bioactive systems exhibit diminishing ion release over time, unstable pH-neutralizing capabilities, or compromised mechanical integrity under prolonged aging. Importantly, published investigations rarely extend beyond six months, leaving considerable uncertainty regarding the long-term performance of bioactive restoratives when subjected to clinically relevant aging conditions.^9^, ^10^, ^11^, ^12^ Moreover, most available studies focus on a single class of bioactive material or evaluate only one functional parameter, such as ion release or mechanical strength, in isolation. Comprehensive long-term investigations integrating ion release, fluoride recharge potential, pH modulation, and mechanical reliability across different bioactive materials are scarce. Addressing this gap is essential to understand the long-term benefits of next-generation bioactive materials.

The objective of this study was to compare the 12-month ion release profile, fluoride recharge ability, pH-neutralizing behavior, and mechanical stability of experimental ACP-based resin composites with Activa™ BioACTIVE-Restorative, Cention N, and Surefil one™. This study aimed to determine whether the ACP composite exhibited a more sustained release of calcium, phosphate, and fluoride ions, better re-release of fluoride following recharge, and superior or comparable retention of flexural strength and modulus during extended aging. The null hypothesis proposed that no significant differences existed among the tested materials for any of the evaluated properties.

## 2 Materials and methods

### 2.1 Study design

This in vitro comparative experimental study was conducted between January 2024 and January 2025 at the Department of Conservative Dentistry and Endodontics. As the study did not involve human participants or animal experimentation, ethical approval was waived in accordance with the 2017 guidelines of the Indian Council of Medical Research (ICMR) 2017 guidelines for biomedical research.

### 2.2 Materials and experimental groups

Four restorative materials were evaluated in this study: an experimental resin composite and three commercially available bioactive restorative materials (Activa™ BioACTIVE-Restorative, Cention N, and Surefil one™). An experimental ACP-based composite was fabricated. The resin matrix consisted of Bis-GMA and TEGDMA in a 60:40 wt ratio. Silanized ACP nanoparticles (average particle size: 80–120 nm) were incorporated at 10 wt %. ACP particles were synthesized via a wet chemical precipitation method, followed by drying and milling to obtain nanoscale particles. Prior to incorporation, the particles were silanized using γ-methacryloxypropyltrimethoxysilane to enhance the filler–matrix bonding. The composite paste was prepared using a mechanical mixer under controlled conditions to ensure homogeneous dispersion of the fillers. The resulting paste was stored in a light-proof container at room temperature until further use. Three commercial bioactive restorative materials were used, according to the manufacturer's instructions. The detailed material compositions, curing modes, and manufacturer details are provided in Supplementary Table S1.

### 2.3 Specimen allocation and sample size justification

As flexural strength and flexural modulus testing are destructive, the specimens were tested only once at each time point. Therefore, separate bar specimens were prepared at each evaluation time point. In contrast, ion release and pH analyses require longitudinal evaluation of the same specimens over time.

The sample size was determined a priori using G*Power 3.1 (Heinrich Heine University, Düsseldorf, Germany) for a mixed-model ANOVA design with four material groups. Assuming a large effect size (f = 0.50), α = 0.05, and power (1–β) = 0.80, a minimum of eight specimens per group was required. To ensure adequate power and account for specimen loss during long-term aging, ten specimens per group were allocated to each testing condition.

Accordingly, a total of 40 specimens per material were prepared: 10 specimens for longitudinal ion release, pH modulation, and fluoride recharge and 30 specimens for flexural testing (10 specimens each at baseline, 6 months, and 12 months).

### 2.4 Specimen preparation and grouping

The specimens were fabricated by a single calibrated operator under standardized laboratory conditions (23 ± 1^0^C and 50 ± 5% relative humidity). Two independent specimen sets with different geometries were prepared, as follows:

Set 1: Ion release, pH modulation, and fluoride recharge: Disc-shaped specimens (10 mm diameter × 2 mm thickness; n = 10 per material) were prepared for the evaluation of calcium (Ca^2+^), phosphate (PO_4_^3−^), and fluoride (F^−^) ion release, pH changes, and fluoride recharge. These specimens were evaluated longitudinally over 365 d and were not subjected to mechanical testing.

Set 2: Flexural strength and flexural modulus: Bar-shaped specimens (25 × 2 × 2 mm; n = 30 per material) were prepared for mechanical testing. The flexural strength and flexural modulus were evaluated at three time points: baseline (24 h), 6 months, and 12 months, using independent sets of 10 specimens per material at each time point. The specimens tested at one time point were not reused at the subsequent time points.

The light-activated materials were polymerized using a polywave LED curing unit delivering an irradiance of at least 1200 mW/cm^2^ for 20 s per surface. Cention N and Surefil one™ were primarily allowed to self-cure, with optional light activation applied for Activa™ BioACTIVE-Restorative and Surefil one™ in accordance with the manufacturer's recommendations. After polymerization, all specimens were finished using sequential 800- and 1200-grit silicon carbide abrasive papers under water irrigation, ultrasonically cleaned for 5 min, air-dried, and stored in distilled water at 37^0^C for 24 h prior to the baseline testing.

### 2.5 Aging protocol

Long-term aging was performed by storing each specimen individually in 10 mL of artificial saliva prepared according to the ISO/TS 16506:2018 specifications. All specimens were maintained at 37^0^C throughout the aging period to simulate intraoral temperature conditions. The storage medium was refreshed weekly to prevent ionic saturation and to maintain consistent diffusion gradients. The specimens were kept under static conditions between the solution changes, and no mechanical or magnetic agitation was applied during the storage period.

To simulate the long-term thermal stresses encountered in the oral environment, thermocycling was performed at 12-month intervals using alternating water baths at 5^0^C and 55^0^C, with a dwell time of 30 s and a transfer time of 10 s, for a total of 10,000 cycles. This regimen corresponds to approximately one year of clinical thermal aging. Specimens designated for flexural testing were stored under identical conditions and retrieved only at their respective testing intervals.

### 2.6 Ion release and pH evaluation

Ion release measurements were performed at 1, 30, 180, and 365 d. At each time point, the specimens were transferred to fresh artificial saliva and the pH of the storage medium was recorded using a calibrated benchtop pH meter. Calcium and phosphate ion concentrations were quantified by inductively coupled plasma optical emission spectrometry (ICP-OES; Optima 8000, PerkinElmer, USA). Prior to the analysis, calibration curves were established using standard solutions of known concentrations. Detection limits were 0.01 ppm for calcium and 0.02 ppm for phosphate. All measurements were performed in triplicates to ensure analytical reliability. Fluoride ion release was measured using a fluoride ion-selective electrode (Orion 9609BNWP, Thermo Scientific, USA) after the addition of total ionic strength adjustment buffer (TISAB III) in a 1:1 ratio to maintain constant ionic strength and pH. Calibration was performed using fluoride standards ranging from 0.1 to 10 ppm. The cumulative ion release values were normalized to the specimen surface area and expressed as μg/cm^2^.

### 2.7 Fluoride recharge evaluation

Fluoride recharge was evaluated at the 6-month time point using a single standardized recharge protocol. The disc specimens were exposed to a neutral sodium fluoride gel containing 5000 ppm fluoride for 5 min, rinsed thoroughly with distilled water, and returned to fresh artificial saliva. Fluoride re-release was subsequently measured every week for 30 days using a fluoride ion-selective electrode under standardized conditions, and the cumulative fluoride release was calculated over the evaluation period. This protocol was designed to assess the intrinsic fluoride uptake and re-release capacity rather than the effects of repeated recharge cycles. Fluoride uptake in this protocol is expected to occur primarily through surface adsorption and superficial diffusion into the outer resin matrix, rather than incorporation into the bulk material structure.

### 2.8 Mechanical and physical property evaluation

The flexural strength and modulus were determined using a three-point bending test in accordance with ISO 4049, with a support span of 20 mm and crosshead speed of 1 mm/min. The load was applied at the center of the specimen until fracture, and the maximum load at the fracture was recorded for the calculations. Both parameters were derived from the same specimen, and the flexural modulus was calculated from the linear elastic region of the load–deflection curve, and the flexural strength was calculated from the maximum load at fracture. All mechanical tests were performed at baseline (24 h) and at 6 and 12 months by a blinded examiner.

### 2.9 Statistical analysis

Statistical analyses were performed using the SPSS software (version 28.0; IBM Corp., Armonk, NY, USA). Data distribution was assessed using the Shapiro–Wilk test, and all variables satisfied the assumptions of normality. For repeated-measures data, sphericity was evaluated using Mauchly's test, and Greenhouse–Geisser corrections were applied when violations were detected. Calcium (Ca^2+^), phosphate (PO_4_^3−^), fluoride (F^−^) ion release, pH changes, and fluoride re-release following recharge were analyzed using mixed-model ANOVA, with material as the between-subject factor and time as the within-subject factor. Bonferroni-adjusted post hoc tests were used for pairwise comparisons. Effect sizes were calculated using partial eta squared (η^2^). The flexural strength and flexural modulus were analyzed using mixed-model ANOVA with material and aging time as fixed factors. Because flexural testing is destructive, independent specimen sets were used at each time point, and no repeated measures were applied for mechanical testing. Tukey's post-hoc test was used for multiple comparisons. The flexural strength reliability was evaluated using Weibull analysis, with the Weibull modulus (m) and characteristic strength calculated using maximum likelihood estimation and 95% confidence intervals. Statistical significance was set at p < 0.05 for all analyses.

## 3 Results

### 3.1 Ion release and pH modulation

The cumulative release of calcium (Ca^2+^), phosphate (PO_4_^3−^), fluoride (F _4_), and corresponding pH changes over 365 days are presented in Table 1. All the materials exhibited an initial burst release on day 1, followed by a progressive decline over time. Mixed-model ANOVA demonstrated statistically significant main effects of material and time as well as significant material × time interactions for all ions and pH values (p = 0.001; Table 2). The effect sizes were large (η^2^ = 0.19–0.69), indicating substantial differences among the materials.

**Table 1:** Cumulative ion release (Ca^2+^, PO_4_^3−^, F^−^ in μg/cm^2^) and pH values of the restorative materials over 365 days in artificial saliva.

Material	Time Point	Ca^2+^ Release (μg/cm^2^)	PO_4_^3−^ Release (μg/cm^2^)	F^−^ Release (μg/cm^2^)	pH of Solution
**Experimental** **ACP Composite (N = 10)**	Day 1	23.40 ± 1.84	23.80 ± 5.49	0.29 ± 0.14	6.61 ± 0.53
	Day 30	3.90 ± 1.20	5.80 ± 2.04	0.19 ± 0.12	7.20 ± 0.16
	Day 180	1.19 ± 0.49	1.80 ± 0.70	0.08 ± 0.06	7.13 ± 0.14
	Day 365	0.98 ± 0.23	1.24 ± 0.54	0.04 ± 0.05	7.12 ± 0.12
**Activa^TM^ BioACTIVE (N = 10)**	Day 1	16.30 ± 4.16	13.60 ± 3.37	7.50 ± 2.42	7.62 ± 0.15
	Day 30	8.90 ± 3.14	8.10 ± 2.69	4.70 ± 2.00	7.82 ± 0.34
	Day 180	4.10 ± 1.97	5.40 ± 2.32	3.00 ± 1.89	7.73 ± 0.31
	Day 365	3.95 ± 1.65	4.34 ± 1.85	2.65 ± 1.14	7.72 ± 0.25
**Cention N (N = 10)**	Day 1	24.40 ± 4.81	18.80 ± 4.32	13.30 ± 3.59	8.53 ± 0.25
	Day 30	13.60 ± 4.20	17.20 ± 4.26	8.90 ± 3.03	8.32 ± 0.21
	Day 180	8.40 ± 2.50	7.30 ± 2.21	6.60 ± 2.12	7.79 ± 0.34
	Day 365	7.56 ± 1.75	6.85 ± 2.11	6.15 ± 1.45	7.50 ± 0.25
**Surefil one™ (N = 10)**	Day 1	3.30 ± 1.16	2.50 ± 0.97	23.10 ± 5.04	7.25 ± 0.17
	Day 30	1.08 ± 0.53	2.13 ± 1.02	10.40 ± 2.99	7.06 ± 0.15
	Day 180	0.50 ± 0.25	0.46 ± 0.25	6.70 ± 1.42	7.01 ± 0.12
	Day 365	0.45 ± 0.18	0.35 ± 0.22	6.50 ± 1.35	7.01 ± 0.10

Values are presented as mean ± standard deviation (SD), N = 10 specimens per material. Ion release values represent cumulative release normalized to specimen surface area (μg/cm^2^), Measurements were performed in artificial saliva at 37 °C with weekly medium refresh.

**Table 2:** Mixed-model ANOVA and post-hoc pairwise comparisons for calcium (Ca^2+^), phosphate (PO_4_^3−^), fluoride (F^−^) ion release and pH.

Parameters	Source of variation	F-value	p-value	η^2^
Calcium (Ca^2+^)	Time	462.63	0.001*	0.44
	Material	75.03	0.001*	0.33
	Time x Material	49.59	0.001*	0.14
Phosphate (PO_4_^3−^)	Time	155.72	0.001*	0.33
	Material	83.79	0.001*	0.35
	Time x Material	31.89	0.001*	0.20
Fluoride (F^−^)	Time	127.45	0.001*	0.19
	Material	88.70	0.001*	0.54
	Time x Material	32.48	0.001*	0.14
pH	Time	6.23	0.003*	0.02
	Material	98.14	0.001*	0.69
	Time x Material	14.62	0.001*	0.11

B. Post-hoc pairwise comparisons (Bonferroni-adjusted)								
Pairwise comparison	Ca^2+^ Release		PO_4_^3−^ Release	F^−^ Release		pH		
	t-value	p-value	t-value	p-value	t-value	p-value	t-value	p-value
Experimental ACP Composite vs. ACTIVA™ BioACTIVE-Restorative	−0.29	1	1.74	0.539	−5.69	0.001*	−9.09	0.001*
Experimental ACP Composite vs. Cention N	−6.43	0.001*	−4.82	0.001*	−10.98	0.001*	−15.09	0.001*
Experimental ACP Composite vs. Surefil one™	8.48	0.001*	10.67	0.001*	−15.41	0.001*	−1.55	0.78
ACTIVA™ BioACTIVE-Restorative vs. Cention N	−6.14	0.001*	−6.57	0.001*	−5.29	0.001*	−5.99	0.001*
ACTIVA™ BioACTIVE-Restorative vs. Surefil one™	8.77	0.001*	8.92	0.001*	−9.72	0.001*	7.54	0.001*
Cention N vs. Surefil one™	14.91	0.001*	15.49	0.001*	−4.43	0.001*	13.54	0.001*

Mixed-model ANOVA with material (between-subjects) and time (within-subjects/repeated measures) factors, Partial η^2^ = partial eta-squared (effect size), *p < 0.05 indicates statistical significance, Post-hoc tests are Bonferroni-adjusted for multiple comparisons, Data derived from N = 10 specimens per material.

The ACP composite exhibited the highest early Ca^2+^ and PO_4_^3−^ release at Day 1, followed by a marked reduction at subsequent time points, with negligible fluoride release throughout the study period. Activa™ BioACTIVE-Restorative demonstrated a sustained release of calcium, phosphate, and fluoride ions across the evaluation period, accompanied by moderately alkaline pH values. Cention N showed a sustained multi-ion release, particularly fluoride, and maintained the highest alkaline pH among the tested materials. Surefil one™ released minimal calcium and phosphate, but exhibited significantly higher fluoride release than the other materials at multiple time points, while maintaining near-neutral pH values. Bonferroni-adjusted post-hoc comparisons confirmed statistically significant differences in ion release among the materials at most evaluated time points (p < 0.05; Table 2).

### 3.2 Mechanical properties

The flexural strength and modulus results are listed in Table 3. Mixed-model ANOVA revealed a highly significant main effect of material on both flexural strength and flexural modulus (p = 0.001), with large effect sizes (η^2^ = 0.64, flexural strength and 0.76 for flexural modulus, respectively). The aging time produced a statistically significant reduction in the mechanical properties of all materials.

**Table 3:** Mixed-model ANOVA results and Tukey-adjusted post-hoc pairwise comparisons for flexural strength and flexural modulus of the restorative materials at baseline, 6 months, and 12 months of aging.

Parameters	Source of variation	F-value	p-value	η^2^
Flexural strength (MPa)	Aging Time	252.99	0.001*	0.03
	Material	23.41	0.001*	0.64
	Aging Time x Material	3.24	0.007*	0
Flexure modulus (GPa)	Aging Time	370.67	0.001*	0.07
	Material	55.36	0.001*	0.76
	Aging Time x Material	11.03	0.001*	0.01

Post-hoc pairwise comparisons (Tukey-adjusted)				
Pairwise comparison	Flexure strength (MPa)		Flexure modulus (GPa)	
	t-value	p-value	t-value	p-value
Experimental ACP Composite vs. ACTIVA™ BioACTIVE-Restorative	−2.49	0.104	−2.88	0.04*
Experimental ACP Composite vs. Cention N	−5.56	0.001*	−7.1	0.001*
Experimental ACP Composite vs. Surefil one™	−7.8	0.001*	−12.09	0.001*
ACTIVA™ BioACTIVE-Restorative vs. Cention N	−3.06	0.025*	−4.22	0.001*
ACTIVA™ BioACTIVE-Restorative vs. Surefil one™	−5.3	0.001*	−9.2	0.001*
Cention N vs. Surefil one™	−2.24	0.188	−4.98	0.001*

Mechanical testing was performed on independent specimens at each time point (baseline, 6 months, 12 months) due to the destructive nature of flexural testing, Mixed-model ANOVA with material and aging time as fixed factors, Partial η^2^ = partial eta-squared (effect size), Values < 0.05 denotes statistical significance.

Surefil one™ and Cention N demonstrated the highest flexural strength and modulus values at all time points, with no statistically significant differences between them (p > 0.05). Activa™ BioACTIVE-Restorative exhibited an intermediate mechanical performance. The ACP composite showed significantly lower flexural strength and modulus than Cention N and Surefil one™ (p = 0.001). Reductions in the flexural modulus were more pronounced than reductions in the flexural strength over time.

### 3.3 Flexural strength reliability (Weibull analysis)

**Fig. 1:**
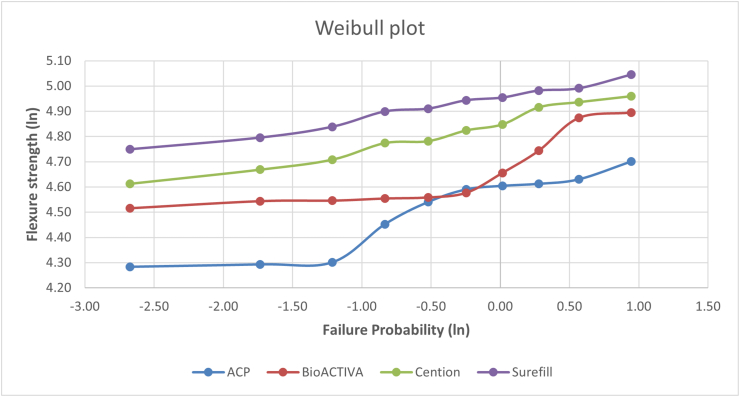
Weibull probability plots illustrating the flexural strength reliability of ACP Composite, Activa™ BioACTIVE-Restorative, Cention N, and Surefil one™ at baseline, 6 months, and 12 months of aging. Steeper slopes indicate higher Weibull modulus (m) values and greater mechanical reliability, while leftward shifts reflect lower characteristic strength. Progressive reductions in slope with aging demonstrate increased variability in flexural strength over time. Weibull parameters were calculated using maximum likelihood estimation based on the flexural strength data obtained from independent specimens tested at each time point. The Weibull modulus (m) represents the variability of strength, with higher values indicating a more consistent and predictable mechanical performance.

The Weibull parameters describing the flexural strength reliability at baseline, 6 months, and 12 months are presented in Table 4, and the corresponding Weibull plots are illustrated in Fig. 1. The Weibull modulus values decreased over time for all materials, indicating an increased variability in strength with aging. Surefil one™ and Cention N demonstrated the highest Weibull modulus values at all time points, whereas the ACP composite exhibited the lowest values. Activa™ BioACTIVE-Restorative showed intermediate reliability, with moderate changes over time.

**Table 4:** Weibull modulus (m) values describing flexural strength reliability of restorative materials at baseline, 6 months, and 12 months.

Material	Baseline (CI at 95%)	6 months (CI at 95%)	12 months (CI at 95%)
**Experimental ACP Composite**	6.63 (4.8-8.5)	6.24 (4.2-8.3)	5.78 (3.5-7.2)
**Experimental ACP Composite**	7.65 (6.3-9.0)	7.12 (5.8-8.4)	6.98 (5.5-8.2)
**Cention N**	9.25 (7.9-10.6)	8.92 (7.5- 9.8)	8.65 (7.1-9.5)
**Surefil one™**	9.45 (8.6-10.3)	8.34 (7.5- 10.1)	8.15 (6.8-9.6)

Weibull modulus (m) represents flexural strength reliability; higher m values indicate lower variability and greater structural consistency, a progressive reduction in Weibull modulus over time indicates decreased mechanical reliability due to aging, Weibull parameters were estimated using maximum likelihood estimation based on flexural strength data, CI: Confidence Interval.

### 3.4 Fluoride recharge and re-release

Cumulative fluoride re-release following a single 5000-ppm NaF recharge at six months is presented in Table 5. Mixed-model ANOVA revealed statistically significant effects of material, time, and material × time interaction on fluoride re-release (p = 0.001; Table 6), with material accounting for the largest proportion of the variance (η^2^ = 0.72). Surefil one™ demonstrated the highest cumulative fluoride re-release over the 30-day evaluation period, followed by Cention N and Activa™ BioACTIVE-Restorative, whereas the ACP composite showed minimal re-release. All materials exhibited an initial Day-1 increase in fluoride release after recharge, followed by a gradual decline over time.

**Table 5:** Cumulative fluoride re-release (μg/cm^2^) over 30 days following a single 5000 ppm NaF gel recharge at 6 months (Mean ± SD, n = 10 per group).

Material	Day 1 post-recharge	Day 7 post-recharge	Day 14 post-recharge	Cumulative at Day 30
Experimental ACP Composite	0.48 ± 0.15	0.35 ± 0.12	0.06 ± 0.05	0.65 ± 0.18
ACTIVA™ BioACTIVE-Restorative	7.10 ± 1.50	5.20 ± 1.10	3.65 ± 1.64	9.50 ± 2.00
Cention N	11.20 ± 2.40	8.30 ± 1.80	6.75 ± 1.85	14.80 ± 3.10
Surefil one™	16.80 ± 3.00	12.50 ± 2.20	7.60 ± 2.35	22.00 ± 4.00

Values represent mean ± standard deviation of cumulative fluoride re-release (μg/cm^2^, normalized to specimen surface area) measured over 30 days following a standardized single recharge with 5000 ppm sodium fluoride gel at the 6-month time point. n = 10 specimens per material.

**Table 6:** Mixed-model ANOVA results and Bonferroni-adjusted post-hoc pairwise comparisons for cumulative fluoride re-release following 5000 ppm NaF recharge at 6 months.

Parameters	Source of variation	F-value	p-value	Partial η^2^
Fluoride re-release	Time	212.59	0.001*	0.13
	Material	35.47	0.001*	0.72
	Time x Material	5.14	0.001*	0.23

Post-hoc pairwise comparisons (Bonferroni-adjusted)		
Pairwise comparison	Fluoride re-release	
	t-value	p-value
Experimental ACP Composite vs. ACTIVA™ BioACTIVE-Restorative	−3.19	0.001*
Experimental ACP Composite vs. Cention N	−6.46	0.001*
Experimental ACP Composite vs. Surefil one™	−8.34	0.001*
ACTIVA™ BioACTIVE-Restorative vs. Cention N	−4.06	0.025*
ACTIVA™ BioACTIVE-Restorative vs. Surefil one™	−5.93	0.001*
Cention N vs. Surefil one™	−4.24	0.001*

Mixed-model ANOVA with material as the between-subjects factor and post-recharge time (Day 1, 7, 14, 30) as the within-subjects (repeated-measures) factor, Partial η^2^ = partial eta-squared (effect size), *p < 0.05 denotes statistical significance, Post-hoc tests are Bonferroni-adjusted for multiple comparisons, Data derived from n = 10 specimens per material.

### 3.5 Summarized results

**Fig. 2:**
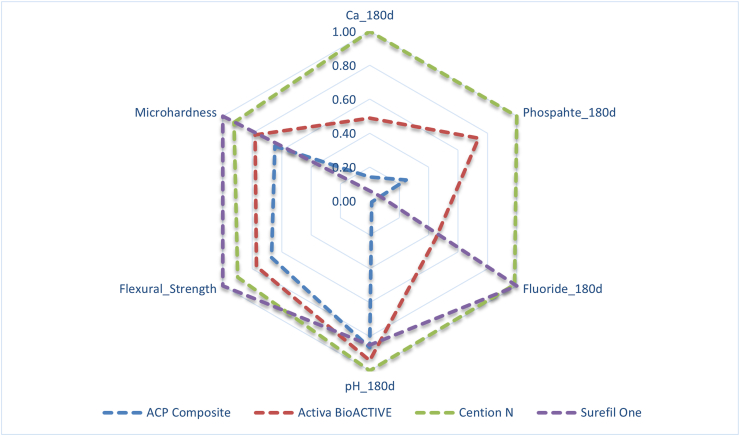
Radar plot illustrating the normalized comparative performance of experimental ACP Composite, Activa™ BioACTIVE-Restorative, Cention N, and Surefil one™ at 180 days. Parameters include calcium release, phosphate release, fluoride release, pH modulation, flexural strength, and microhardness. Values were normalized to allow inter-material comparison across different measurement scales. Larger radial extension indicates higher relative performance for the respective parameter.

The radar plot demonstrated distinct material-specific performance profiles at 180 d. Cention N showed the most balanced overall performance, with high calcium and phosphate release, strong alkalizing capacity, and favorable mechanical properties. Surefil one™ exhibited the highest fluoride release and superior mechanical performance, but limited calcium and phosphate release. The Activa™ BioACTIVE-Restorative demonstrated moderate multi-ion release and intermediate mechanical characteristics. The experimental ACP composite showed relatively higher calcium release compared with fluoride but demonstrated lower mechanical properties and limited long-term ion sustainability. The graphical representation highlights the material-dependent divergence between bioactivity and mechanical stability (Fig. 2).

## 4 Discussion

The present study compared the long-term ion release, pH modulation, mechanical stability, and structural reliability of an experimental ACP-based bioactive resin composite with three commercial bioactive restoratives (Activa™ BioACTIVE-Restorative, Cention N, and Surefil one™) over a one year in vitro. The present study was designed to provide a multidimensional evaluation of these properties over an extended aging period.

The study found distinct bioactive profiles: the ACP composite demonstrated a rapid initial burst of calcium and phosphate release followed by a marked decline, while Cention N exhibited superior multi-ion release and alkalization, Activa™ BioACTIVE-Restorative provided a balanced intermediate performance, and Surefil one™ excelled in sustained fluoride delivery. These findings support the concept that contemporary bioactive restorative materials are not functionally equivalent; instead, they exhibit material-specific ion release patterns and mechanical behaviors depending on their filler composition and setting chemistry.

### 4.1 Calcium and phosphate release behavior

The experimental ACP-based resin composite exhibited a pronounced initial burst of Ca^2+^ and PO_4_^3−^ release at 1 d, with cumulative values declining sharply thereafter and negligible F^−^ output over 365 days, alongside near-neutral pH. This kinetic profile stems from the high solubility and thermodynamic instability of amorphous calcium phosphate nanoparticles, which rapidly dissolve in aqueous environments to supersaturate with ions for early remineralization but subsequently deplete or convert to stable apatite phases, thereby limiting long-term bioactivity. Previous studies on ACP-containing composites have similarly reported an initial burst release followed by a sharp decline, which is attributed to rapid surface dissolution and subsequent depletion of readily available ACP particles.^13^, ^14^, ^15^

In contrast, Activa™ BioACTIVE-Restorative and Cention N demonstrated more sustained multi-ion release profiles. This difference may be explained by the distinct chemistry of the materials. Activa incorporates a bioactive glass component within a resin-based matrix, which undergoes gradual surface reactions leading to prolonged ion release.^7^ Cention N, as an alkasite material, contains alkaline fillers designed to release fluoride, calcium, and hydroxyl ions over time, contributing to its sustained ion release and alkaline pH environment.^9^ Kasraei et al.^16^ reported the highest phosphate ion release with Cention N followed by Activa™ BioACTIVE-Restorative over six months. Ruengrungsom et al.^17^ reported that Activa™ BioACTIVE-Restorative showed the highest phosphate ion release, whereas Cention N displayed the greatest recharge ability during a 14-day study period.

### 4.2 Fluoride release and recharge behavior

The fluoride release patterns differed markedly among the materials. Surefil one™ demonstrated a bioactivity profile predominantly characterized by fluoride release, achieving the highest cumulative fluoride levels among the tested materials, while exhibiting minimal calcium and phosphate ion release. This behavior is consistent with its giomer-like chemistry, incorporating pre-reacted glass (PRG) fillers that function as stable fluoride reservoirs rather than sources of sustained alkaline ion release.^18^^,^^19^ Consequently, the surrounding medium remained near neutral pH, consistent with previous literature indicating that fluoride-focused restorative systems primarily act as fluoride reservoirs and lack pronounced alkalizing capacity.^20^

Cention N also exhibited sustained fluoride release and moderate recharge capability, consistent with the presence of ytterbium fluoride and calcium fluorosilicate fillers in its composition. The Activa™ BioACTIVE-Restorative showed intermediate fluoride release and recharge behavior, likely reflecting the combined effects of its resin matrix and bioactive glass fillers. In contrast, the ACP composite demonstrated negligible fluoride release and minimal recharge capacity, which was expected given the absence of fluoride-containing fillers in its formulation. This finding confirms that fluoride recharge behavior is primarily dependent on the presence of fluoride-reactive phases rather than general material porosity or water uptake.

### 4.3 pH modulation and alkalizing capacity

The ability of restorative materials to neutralize acidic environments is a key factor in their anti-cariogenic potential. In this study, Cention N maintained the most alkaline pH values across all time points, which can be attributed to the release of hydroxyl ions from the alkaline fillers. The Activa™ BioACTIVE-Restorative demonstrated moderately alkaline conditions, consistent with the buffering action of its bioactive glass component.

The ACP composite maintained near-neutral pH despite its high early calcium and phosphate release. This observation may be explained by the dissolution behavior of ACP, which primarily releases calcium and phosphate ions without generating significant concentrations of hydroxyl ions. Surefil one™ also maintained a near-neutral pH, reflecting its fluoride-dominant rather than alkalizing ion-release profile.

### 4.4 Mechanical performance and structural reliability

Surefil one ™ showed the highest mechanical performance, with superior flexural strength and elastic modulus maintained throughout aging, supported by its reinforced resin matrix and effective filler–matrix integration. A high Weibull modulus indicates an excellent reliability and low strength variability.^21^^,^^22^ Cention N exhibited high flexural strength and consistent Weibull reliability over time, which was attributed to the high filler loading and homogeneous polymerization that minimized internal stresses. These results align with prior findings on the stable performance of self-curing alkasite materials.^11^^,^^23^^,^^24^

Activa™ BioACTIVE-Restorative displayed intermediate performance, with a flexural strength better than that of the ACP composite but lower than that of alkasite- and giomer-based materials. Aging caused modest declines in strength and stiffness, likely from hydrolytic degradation.^25^ Clinical studies still report acceptable one-year outcomes in Class II restorations.^26^ The experimental ACP-based composite had the lowest performance, with significant post-aging reductions in flexural strength and elastic modulus owing to matrix plasticization, filler–matrix interface weakening, and ACP phase dissolution, creating microstructural voids. Weibull analysis showed lower reliability and higher fracture risk, consistent with earlier reports on ACP resins.^13^^,^^14^^,^^27^

### 4.5 Influence of long-term aqueous aging

Long-term aqueous aging produced measurable reductions in the mechanical properties of all materials, although the extent of degradation varied according to the material composition and polymerization chemistry. Water sorption is known to induce plasticization of the resin matrix, hydrolysis of silane coupling agents, and gradual debonding at the filler–matrix interface, ultimately reducing stiffness and strength over time. In addition, ion-releasing materials may experience structural changes associated with the dissolution of reactive filler phases, which can further compromise their mechanical integrity. Previous studies have reported similar aging-related reductions in flexural properties for resin-based and bioactive restorative materials, attributing these effects to hydrolytic degradation and filler dissolution processes.^11^ The relatively stable mechanical behavior observed in Cention N and Surefil one™ suggests that higher filler loading, more stable polymer networks, and reduced solubility of their reactive phases may contribute to improved resistance against long-term aqueous degradation.

### 4.6 Clinical implications and material-specific bioactivity profiles

The distinct ion release and mechanical profiles observed in this study suggest that bioactive restorative materials should not be considered as interchangeable. Instead, each material demonstrated a characteristic bioactivity profile based on its filler composition and chemical structure. ACP-based composites may be more suitable in situations where rapid calcium and phosphate release is desired, whereas fluoride-focused or alkasite materials may provide sustained fluoride release and alkalizing effects.

### 4.7 Strengths and limitations

A key strength of the present study was the integrated evaluation of ion release, fluoride recharge, pH modulation, and mechanical aging within a single experimental framework. This approach provides a more comprehensive understanding of the long-term behavior of bioactive restorative materials than studies focusing on isolated properties. However, this study had several limitations. The study was conducted under in vitro conditions, which cannot fully replicate the complex biological and mechanical environments of the oral cavity. Additionally, the aging protocol involved static immersion rather than dynamic salivary flow, and the evaluation period was limited to one year. Future studies incorporating cyclic loading, biofilm models, and longer aging periods would provide further insights into the clinical performance of these materials.

## 5 Conclusions

Within the limitations of this in vitro study, the evaluated materials exhibited distinct material-dependent bioactive and mechanical behaviors after 12 months of aging. The experimental ACP composite showed pronounced early calcium phosphate release but reduced long-term mechanical stability. Cention N demonstrated sustained multi-ion release, the greatest alkalizing effect, and consistently high mechanical performance. Activa™ BioACTIVE-Restorative provided balanced ion release with intermediate strength, whereas Surefil one™ showed fluoride-dominant behavior combined with the highest mechanical reliability. These results indicate that bioactive restorative materials are not functionally equivalent and that their clinical performance may depend on the balance between ion release and long-term mechanical stability. Further clinical studies are required to confirm the long-term relevance of these findings.

## Patient's/guardian's consent

None of the patients was included in this study. Hence, patient or guardian consent was not required.

## Ethical approval

As no human or human tissue was involved in the study, therefore, waiver was given by Institutional Ethical Committee of NITTE (Deemed to be University) AB Shetty Memorial Institute of Dental Sciences (ETHICS/ABSMIDS/502/2024).

## Authors’ contributions

Anshuman Shetty contributed to the conceptualization and design of the study, conducted specimen preparation and laboratory experiments, participated in data acquisition, and drafted the initial manuscript.

Mohammed Abdul Saleem contributed to methodological planning, critical input on experimental design, and reviewed the manuscript for important intellectual content.

Ameer Akhil Ahmed Shaik assisted in specimen preparation, data collection, and preliminary data organization.

Mohammed Mustafa contributed to interpretation of results, validation of experimental outcomes, and critical revision of the manuscript.

Rahul Tiwari contributed to study conception and supervision, statistical analysis, interpretation of data, manuscript editing, and final approval of the version to be published.

Tanisha Shetty assisted in laboratory procedures, data entry, and literature review.

All authors have made substantial contributions to the work, approved the final manuscript, and agree to be accountable for all aspects of the work, in accordance with the ICMJE authorship criteria.

## Human participants statement

This study did not involve human participants, human biological samples, or identifiable personal data. Therefore, approval from an institutional ethics committee and informed consent were not required.

## Animal ethics statement

This study did not involve animal subjects or animal-derived materials. Accordingly, approval from an animal ethics committee was not required.

## Sources of funding

This research did not receive any specific grants from funding agencies in the public, commercial, or not-for-profit sectors.

## Declaration of competing interest

The authors declare that they have no known competing financial interests or personal relationships that could have appeared to influence the work reported in this paper.
