# Supplementary material for: Long-term ion release, fluoride recharge, pH modulation, and mechanical aging of an experimental ACP-based composite compared with contemporary bioactive restorative materials
Source: J Oral Biol Craniofac Res. 2026 Mar 12;16(3):101431. doi: 10.1016/j.jobcr.2026.101431 (PMC12997216; doi:10.1016/j.jobcr.2026.101431)
Supplement: Multimedia component 1 [file mmc1.docx]

**Supplementary Table S1: Composition and key characteristics of the tested restorative materials.**

| **Material** | **Manufacturer (Headquarters)** | **Curing Mode** | **Key Composition** |
| --- | --- | --- | --- |
| Experimental ACP Composite | Self fabricated | Light-cured | Bis-GMA/TEGDMA resin matrix (60:40 wt%); silanated barium borosilicate reinforcing fillers; 10 wt% silanated amorphous calcium phosphate (ACP) nanoparticles (80–120 nm) |
| Activa^TM^ BioACTIVE-Restorative | Pulpdent Corporation (Watertown, MA, USA) | Dual-cure (light + self-cure) | Bioactive ionic resin matrix; shock-absorbing rubberized resin; bioactive glass fillers (releases Ca²⁺, PO₄³⁻, F⁻ with recharge); no Bis-GMA, no BPA derivatives |
| Cention N | Ivoclar Vivadent AG (Schaan, Liechtenstein) | Dual-cured (self-cure primary; optional light-cure) | Powder-liquid system; ~78.4 wt% inorganic fillers (barium aluminum silicate glass, ytterbium fluoride, calcium fluorosilicate, patented alkaline filler); isofillers; UDMA/DCP/PEA monomers; releases F, Ca, OH⁻ |
| Surefil one^TM^ | Dentsply Sirona (Charlotte, NC, USA) | Dual-cure (self + light activation) | Self-adhesive composite hybrid; patented modified polyacid (MOPOS) for adhesion; glass fillers; resin matrix combining composite and glass ionomer properties; releases fluoride |
